# Prognostic value of platelet count and lymphocyte to monocyte ratio combination in stage IV non-small cell lung cancer with malignant pleural effusion

**DOI:** 10.1371/journal.pone.0200341

**Published:** 2018-07-13

**Authors:** Jeong Uk Lim, Chang Dong Yeo, Hye Seon Kang, Chan Kwon Park, Ju Sang Kim, Jin Woo Kim, Seung Joon Kim, Sang Haak Lee

**Affiliations:** Division of Pulmonology, Department of Internal Medicine, College of Medicine, The Catholic University of Korea, Seoul, Republic of Korea; University of Texas MD Anderson Cancer Center, UNITED STATES

## Abstract

**Introduction:**

A combination of platelet and lymphocyte to monocyte ratio (LMR) (abbreviated as COP-LMR) has been recently evaluated as systemic inflammatory marker for prognostication in lung cancer. While previous study on COP-LMR has evaluated its prognostic value in NSCLC patients who underwent curative resections, the combination of these two markers has not been evaluated in advanced NSCLC yet.

**Objectives:**

In this study, we evaluated the prognostic value of COP-LMR in stage IV NSCLC with malignant pleural effusion under active anticancer treatment.

**Methods:**

Between January 2012 and July 2016, 217 patients with stage IV NSCLC and MPE undergoing active anticancer treatment were selected for evaluation. If patients had both low LMR (< 2.47) and increased platelet (> 30.0 ×10^4^ mm-3), they were assigned to COP-LMR group 2. Patients with one parameter were assigned to COP-LMR group 1. If none, patients were assigned to COP-LMR group 0.

**Results:**

Median overall survival (OS) (P < 0.001), progression free survival (PFS) (P < 0.001) and histological feature (P = 0.003) showed significant differences among COP-LMR groups. For COP-LMR groups 0, 1 and 2, median survival times were 35.9, 14.7 and 7.4 months, respectively, while median progression free times were 19.2, 13.3 and 7.4 months, respectively. Older age, male, low albumin, high CRP and high COP-LMR (0 vs 1, P = 0.021, hazard ratio (HR): 1.822, 95% confidence interval (CI): 1.096–3.027 and 0 vs 2, P = 0.003, HR: 2.464, 95% CI: 1.373–4.421) were independent predictive factors for shorter OS. Age, sex, histology, albumin, or CRP had no significant influence on PFS. High COP-LMR was the significant factor in predicting shorter PFS (0 vs 1, P = 0.116 and 0 vs 2, P = 0.007, HR: 1.902, 95% CI: 1.194–3.028).

**Conclusions:**

A combination of pretreatment LMR and platelet levels can be used to predict short survival in stage IV NSCLC patients who underwent active anticancer treatment.

## Introduction

Lung cancer is a major cause of cancer-related death worldwide [1]. Among lung cancers, non-small cell lung cancer comprises a large proportion [2]. Of all lung cancers, non-small lung cancer (NSCLC) accounts for 85%, with majority of patients initially diagnosed at advanced stage [3]. Despite improvement in treatment modalities, treatment outcomes of patients with advanced lung cancer remain poor, with median survival less than 12 months [3]. Nevertheless, prognosis can vary according to pretreatment patients’ factors. Among patient related factors, host inflammatory response to tumor can contribute significantly to cancer progression by promoting cancer cell proliferation, evasion of immune-surveillance, tumor metastasis and angiogenesis [4, 5], thus playing key roles in survival of patients with various cancers [6].

Reliable pretreatment prognostic factor is vital to treatment of cancer by allowing risk assessment and appropriate choice of treatment modalities. Inflammatory biomarkers reflect host responses to malignant cells. Recent studies have focused on their predictive ability in many cancers [7–9]. More specifically, several notable inflammatory prognostic markers in NSCLC have been evaluated for their prognostic values. Higher neutrophil to lymphocyte ratio (NLR) can predict shorter survival in advanced NSCLC [10, 11]. NLR can also predict postoperative outcome after curative resection [12–14]. Platelet to lymphocyte ratio is also a prognostic marker in advanced NSCLC [15–17]. Moreover, lymphocyte to monocyte ratio (LMR) is an independent prognostic factor in NSCLC [18]. Thrombocytosis also predicts worse outcomes in lung cancer [19]. In addition to single inflammatory biomarkers, combinations of single biomarkers with known predictability have also been evaluated for their prognostic values [12, 20]. A combination of platelet and LMR (abbreviated as COP-LMR) has been recently evaluated as systemic inflammatory marker for prognostication in lung cancer [21]. While previous study on COP-LMR has evaluated its prognostic value in NSCLC patients who underwent curative resections [21], the combination of these two markers has not been evaluated in advanced NSCLC yet. In this study, we evaluated the prognostic value of COP-LMR in stage IV NSCLC with malignant pleural effusion under active anticancer treatment by assessing its predictability for overall survival and disease progression.

## Material and methods

### Patients’ selection

A total of 217 stage IV NSCLC patients were consecutively selected from a cohort of NSCLC patients with malignant pleural effusion (MPE). These cohort patients were diagnosed with NSCLC between January 2012 and July 2016. These patients were enrolled from Seoul St. Mary’s Hospital, Incheon St. Mary’s Hospital, Yeouido St. Mary’s Hospital, Bucheon St. Mary’s Hospital, St. Paul’s Hospital and Uijeongbu St. Mary’s Hospital. They had MPE confirmed either cytologically or by pleural biopsy simultaneously. Inclusion criteria for the present study were as follows: 1) histologically confirmed NSCLC; 2) underwent active anticancer treatment; and 3) all clinical data were available. Exclusion criteria were: 1) small cell lung cancer (SCLC) patients; 2) patients who underwent curative lung resection; 3) patients who had significant infection at the time of diagnosis; 4) patients with underlying hematologic disease.

### Clinical and laboratory data

Clinicopathological data including age, sex, histological feature, tumor stages according to tumor–node–metastasis (TNM) criteria (AJCC criteria 2009), smoking status, first line treatment modality and Eastern Cooperative Oncology Group Performance Status Scale (ECOG PS) were evaluated for all enrolled patients. From laboratory data, white blood cell (WBC) count, hemoglobin, platelet, c-reactive protein (CRP), carcinoembryonic antigen (CEA) and lactate dehydrogenase (LDH) were assessed.

### Positive driver mutation

The patients with positive EGFR mutation and/or ALK translocation at diagnosis were classified into a positive driver mutation subgroup. EGFR mutations were defined as exon 19 deletion or exon 21 point mutations. Other more uncommon EGFR mutation profiles were excluded. Genotyping of EGFR was done using peptide nucleic acid (PNA)-mediated PCR clamping methods, such as the PNAClamp TM EGFR Mutation Detection Kit (PANAGENE, Inc., Daejeon, Korea), using real-time PCR.

Specimens for fluorescence in situ hybridization (FISH) acquired from the enrolled hospitals were prepared simultaneously using a molecular analysis platform and were analyzed over a 3-day period at Yeouido St. Mary s Hospital Central Molecular Laboratory. ALK rearrangement positivity was defined as an isolated red signal or split signal. A minimum two-probe diameter distance was necessary for determination of true positive signal splitting. Positive cases were defined as those with>15% of counted nuclei within tumor cells exhibiting a split signal or isolated red signal. For surgical resection specimens, 100 tumor cells were scored. An ALK FISH split signal rate less than 15% was interpreted as negative.

### Chemotherapy and adverse reactions

Systemic conventional chemotherapy regimens were as follows: docetaxel, gemcitabine, pemetrexed, or paclitaxel combined with carboplatin/cisplatin. For targeted therapy, patients with positive epidermal growth factor receptor (EGFR) mutations were treated with gefitinib, erlotinib and afatinib. Patients with positive anaplastic lymphoma kinase (ALK) translocations were treated with crizotinib.

Chemotherapy related adverse reactions for patients enrolled for this study were checked either at the outpatient clinic or at the time of admissions. If patients experienced grade III or IV adverse reactions, treatment was either delayed or converted to other regimens.

### Overall survival and progression free survival

Using Response Evaluation Criteria in Solid Tumors (RECIST) version 1.1, response to treatment was evaluated by treating physicians and independent radiologists. After completing every two cycles of treatment, computed tomography (CT) was performed. OS was defined as the time duration from the date of diagnosis to the date of death. PFS was defined as the time duration from lung cancer diagnosis to disease progression during 1^st^ line treatment. If patients were dead or lost during the follow up period, they were considered censored.

### Categorization by COP-LMR

Blood samples were acquired at the time of diagnosis of NSCLC. LMR was defined as a ratio of peripheral lymphocyte count to peripheral monocyte count. Optimal cutoff values for LMR and platelet counts were calculated based on receiver operating characteristics (ROC) curve analysis. Survival outcomes were dichotomized by survival (alive or death) in ROC analysis. All study patients’ survival status were assessed in October 2017. For LMR, cutoff point was 2.47 with area under curve (AUC) of 0.632. The point on the ROC curve with minimum distance to the upper left corner indicated a cut-off value of 30.05 ×10^4^ mm^-3^ for platelet counts. The AUC was 0.554. Hence, the optimal cut-off value of platelet counts at 30.0 ×10^4^ mm^-3^ was chosen. COP-LMR was calculated from platelet count and LMR. If patients had both low LMR (< 2.47) and increased platelet (> 30.0 ×10^4^ mm^-3^), they were assigned a score of 2 (COP-LMR group 2). Patients with one of these two abovementioned parameters were assigned a score of 1 (COP-LMR group 1). If none of these parameters were found, patients were assigned a sore of 0 (COP-LMR group 0).

### Defining cutoff values

For other laboratory values entered into univariate analyses on OS and PFS, cutoff points were also calculated from ROC curve analysis to categorize patients into a high or a low group. Laboratory parameters were selected based on their correlations with clinical outcomes in NSCLC [14, 22–25]. Similar to LMR and platelet count, survival outcomes were dichotomized by survival (alive or death) in ROC analysis. Optimal cutoff values for continuous variables were determined, using maximally selected log-rank statistics for time to primary endpoint.

The cut-off values for protein, albumin, LDH and CRP were 6.7g/dL, 3.1 g/dL, 488 IU/L and 2.68 mg/dL, respectively. Cutoff values of hemoglobin was 12.9 mg/d. Hence, the optimal cut-off value of hemoglobin was decided as 13.0 mg/dL.

### Statistical analysis

All statistical analyses were performed using Statistical Package for Social Sciences software program version 18.0 (SPSS Inc., Chicago, IL, USA). Continuous variables are presented as mean or median value with range of values. A receiver operating characteristics (ROC) curve was constructed to estimate optimal cut-off values for continuous variables including LMR. For comparison of categorical variables among three COP-LMR groups, Chi-squared test was performed. One-way analysis of variance (ANOVA) was used to compare continuous variables among three COP-LMR groups after normal distribution status had been confirmed.

Cox regression hazard model univariate analysis was performed to determine the significance of variables for OS and PFS. Survival curve graphs were presented after Kaplan–Meier analysis. OS and PFS are shown as median values. Log-rank test was performed to determine the significance of difference in OS and PFS among the three COP-LMR groups. Statistically significant variables from univariate analyses were entered into Cox proportional hazards regression model for multivariate analysis. For all analyses, a *P* value of less than 0.05 was considered statistically significant.

### Ethics statement

The study was approved by the ethics committee at each center (XC17REDI0069U). The requirement for informed consent was waived due to the retrospective nature of this study.

The names of ethics committees are as follows: Seoul St. Mary’s Hospital, Incheon St. Mary’s Hospital, Yeouido St. Mary’s Hospital, Bucheon St. Mary’s Hospital, St. Paul’s Hospital and Uijeongbu St. Mary’s Hospital.

## Results

### Patients characteristics

A total of 217 NSCLC patients were enrolled in this study. Their median age was 68 years (range, 35–92 years). Among all patients, 120 (55.3%) were males and 187 (86.2%) patients were diagnosed with adenocarcinoma. For 1^st^ line anticancer treatment, 148 (68.2%) patients underwent conventional systemic chemotherapy and 69 (31.8%) patients underwent targeted therapy. Median OS and PFS were 16.2 months (range, 12.8–19.6 months) and 14.2 months (range, 12.2–16.2 months), respectively.

Table 1 shows distribution of clinicopathological parameters of study patients in three categories grouped according to COP-LMR. As shown in Table 1, median OS (*P* < 0.001), PFS (*P* < 0.001) and histological feature (*P* = 0.003) showed significant differences among COP-LMR groups. For COP-LMR groups 0, 1 and 2, median survival times were 35.9, 14.7 and 7.4 months, respectively, while median progression free times were 19.2, 13.3 and 7.4 months, respectively. The proportion of adenocarcinoma was the highest in COP-LMR group 0 (90.6%) and the lowest in COP-LMR group 2 (71.4%). Regarding laboratory features, white blood cell (WBC) level (*P* = 0.002), hemoglobin (*P* < 0.001), platelet (*P* < 0.001), albumin (*P* = 0.003) and CRP (*P*<0.001) showed significant differences among three COP-LMR groups.

**Table 1:** Correlation of COP-LMR with the clinicopathological and laboratory parameters of NSCLC patients.

	COP-LMR = 0 (*N*, %)	COP-LMR = 1 (*N*, %)	COP-LMR = 2 (*N*, %)	p-value*
**Number of patients**	64	104	49	
**Median age, range**	66.4 (37–92)	68.9 (47–90)	68.3 (35–87)	0.552
**Median OS (months)**	35.9	14.7	7.4	<0.001
**Median PFS (months)**	19.2	13.3	7.4	<0.001
**Sex**				0.094
** Male**	30 (46.9)	57 (54.8)	33 (67.3)	
** Female**	34 (53.1)	47 (45.2)	16 (32.7)	
**ECOG**				0.717
** 0 and 1**	53 (82.8)	90 (86.5)	43 (87.8)	
** ≥2**	11 (17.2)	14 (13.5)	6 (12.2)	
**Histologic features**				0.003
** Adenocarcinoma**	58 (90.6)	94 (90.4)	35 (71.4)	
** Squamous**	6 (9.4)	10 (9.6)	14 (28.6)	
**Smoking**				0.139
** Never smoker**	39 (60.9)	59 (56.7)	21 (42.9)	
** Ever smoker**	25 (39.1)	45 (43.3)	28 (57.1)	
**T-factor**				0.622
** T1/T2/T3/T4**	4 (6.2)/ 8 (12.5)/ 6 (9.4)/ 27 (42.2)	3 (2.9)/ 12 (11.5)/ 14 (13.5)/ 37 (35.6)	2 (4.1)/ 7 (14.3)/ 6 (12.2)/ 24 (49)	
**N-factor**				0.226
** N0/N1/N2/N3**	4 (6.2)/ 1 (1.6)/ 15 (23.4)/ 27 (42.2)	3 (2.9)/ 7 (6.7)/ 17 (16.3)/ 37 (35.6)	4 (8.2)/ 2 (4.1)/ 9 (18.4)/ 25 (51)	
**M-factor**				0.294
** M1a/M1b**	26 (46.4)/ 30 (53.6)	45 (52.3)/ 41 (47.7)	28 (60.9)/ 18 (39.1)	
**Treatment modality (1**^**st**^ **line)**				0.494
** Conventional chemotherapy**	40 (62.5)	74 (71.2)	34 (69.4)	
** Targeted therapy**	24 (37.5)	30 (28.8)	15 (30.6)	
**Positive driver mutation**	34 (57.6)	40 (42.6)	16 (34.8)	0.051
**WBC count (x10**^**9**^**/L)**	7411.6±2091.6	9012.9±4580.8	9823.5±3336.2	0.002
**Hemoglobin (g/dL)**	13.6±1.6	13.4±1.6	12.3±1.6	<0.001
**Platelet (per/uL)**	247,690±34,673	297,080±66,894	370,490±72,106	<0.001
**Albumin (g/dL)**	3.0±0.6	2.9±0.7	2.6±0.6	0.003
**CRP (mg/L)**	6.2±11.5	15.4±28.5	27.5±38.3	<0.001
**CEA (ng/mL)**	61.1±167.4	229.2±847.4	108.7±244.8	0.398
**LDH (IU/L)**	500.9±248.0	475.7±150.5	518.6±367.9	0.577

CI: confidence interval; COP-LMR: combination of platelet and lymphocyte to monocyte ratio; CEA: carcinoembryonic antigen; CRP: c-reactive protein; ECOG: Eastern Cooperative Oncology Group; HR: hazard ratio; LDH: lactate dehydrogenase; OS: overall survival; PFS: progression free survival; WBC: white blood cell.

*p-value between COP-LMR

### Association of COP-LMR with overall survival and progression free survival

**Fig 1:**
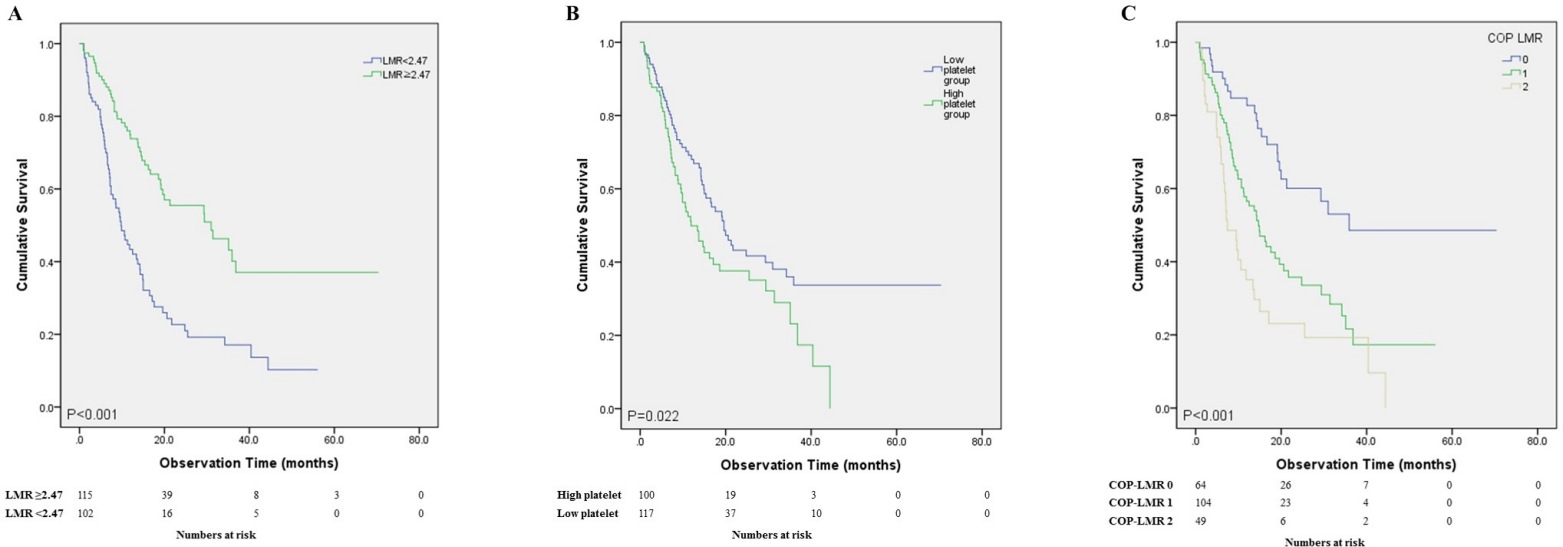
Overall survival of the stage IV NSCLC patients. (A): OS between high and low LMR patients; (B): OS between high and low platelet groups; (C): Overall survival of the stage IV NSCLC patients between the different COP LMR groups. OS showed significant difference between COP LMR groups 0 vs. 1 (P < 0.001) and 1 vs. 2 (P = 0.023). *COP LMR*: *combination of platelet and lymphocyte to monocyte ratio; NSCLC*: *non-small cell lung cancer; OS*: *overall survival*.

Results of univariate analyses for overall survival and progression free survival are shown in Table 2. COP-LMR was a significant factor in predicting OS (0 vs 1: *P* <0.001, hazard ratio (HR): 2.257, 95% confidence interval (CI): 1.393–3.658 and 0 vs 2: *P*<0.001, HR: 3.768, 95% CI: 2.207–6.433) and PFS (0 vs 1: *P* = 0.010, HR: 1.583, 95% CI: 1.118–2.241 and 0 vs 2: *P*<0.001, HR: 2.281, 95% CI: 1.499–3.472). Older age, male and squamous features were also significant predictors for shorter OS and PFS. Of laboratory factors, low LMR (Fig 1A), high platelet count (Fig 1B), low hemoglobin, low albumin and high CRP were significant predictors for shorter OS while low LMR, high platelet count, low albumin and high CRP were significant predictors for shorter PFS.

**Table 2:** Univariate analysis on OS and PFS.

Characteristics	OS			PFS		
	P	HR	95% CI	P	HR	95% CI
Age (≤65/>65)	0.004	1.719	1.184–2.497	0.02	1.420	1.056–1.911
Male	0.001	1.931	1.327–2.811	0.003	1.577	1.170–2.126
Smoking status (ever/never)	0.097	1.166	0.971–1.399	0.068	1.148	0.989–1.332
Histology (adenocarcinoma/squamous cell)	0.005	1.932	1.204–3.103	0.029	1.593	1.049–2.420
Driver mutation (positive/negative)	0.052	1.469	0.996–2.166	0.056	1.354	0.992–1.850
ECOG (0-1/≥2)	0.170	1.197	0.925–1.548	0.835	1.026	0.811–1.297
First line treatment (1^st^ line) (conventional chemotherapy/ targeted therapy)	0.138	1.355	0.906–2.026	0.442	1.132	0.825–1.551
M stage (M1a/M1b)	0.266	1.230	0.843–1.849	0.323	1.177	0.852–1.626
Hemoglobin, g/dL (<13/≥13)	0.011	1.597	1.112–2.294	0.237	1.198	0.888–1.616
Platelet, per uL (<300,000/≥300,000)	0.023	1.523	1.059–2.191	0.010	1.485	1.098–2.008
Protein, g/dL (<6.7/≥6.7)	0.180	1.392	0.968–2.001	0.807	1.098	0.815–1.478
LMR (<2.47/≥2.47)	<0.001	2.487	1.717–3.602	0.002	1.607	1.193–2.165
Albumin, g/dL (<3.1/≥3.1)	<0.001	2.813	1.800–4.395	0.002	1.955	1.276–2.994
LDH, IU/L (<488/≥488)	0.316	1.332	0.918–1.933	0.587	1.089	0.801–1.481
CRP, mg/dL (<2.68/≥2.68)	0.001	2.204	1.498–3.243	0.005	1.553	1.145–2.106
COP-LMR	<0.001			<0.001		
COP-LMR 0	-	1	Referent	-	1	Referent
COP-LMR 1	0.001	2.257	1.393–3.658	0.010	1.583	1.118–2.241
COP-LMR 2	<0.001	3.768	2.207–6.433	<0.001	2.281	1.499–3.472

CI: confidence interval; COP-LMR: combination of platelet and lymphocyte to monocyte ratio; CRP: c-reactive protein; ECOG: Eastern Cooperative Oncology Group; HR: hazard ratio; LDH: lactate dehydrogenase; LMR: lymphocyte to monocyte ratio; OS: overall survival; PFS: progression free survival.

Significant variables from univariate analysis were assessed by multivariate analysis. Histology and hemoglobin had no significant influence on OS. Older age, male, low albumin, high CRP and high COP-LMR (0 vs 1, *P* = 0.021, HR: 1.822, 95% CI: 1.096–3.027 and 0 vs 2, *P* = 0.003, HR: 2.464, 95% CI: 1.373–4.421) were independent predictive factors for shorter OS. Age, sex, histology, albumin, or CRP had no significant influence on PFS. High COP- LMR was the significant factor in predicting shorter PFS (0 vs 1, *P* = 0.116, and 0 vs 2, *P* = 0.007, HR: 1.902, 95% CI: 1.194–3.028). Results are shown in Table 3.

**Table 3:** Multivariate analysis on OS and PFS.

Characteristics	OS			PFS		
	P	HR	95% CI	P	HR	95% CI
Age (≤65/>65)	0.049	1.497	1.002–2.235	0.120	1.289	0.922–1.803
Male	0.027	1.620	1.057–2.481	0.059	1.385	0.988–1.941
Histology (adenocarcinoma/squamous cell)	0.766	1.092	0.610–1.955	0.693	1.108	0.665–1.847
Hemoglobin, g/dL (<13/≥13)	0.317	1.245	0.810–1.914			
Albumin, g/dL (<3.1/≥3.1)	0.035	1.724	1.039–2.861	0.091	1.478	0.939–2.326
CRP, mg/dL (<2.68/≥2.68)	0.011	1.713	1.130–2.599	0.138	1.289	0.922–1.803
COP-LMR (0,1,2)	0.009			0.025		
COP-LMR 0	-	1	Referent	-	1	Referent
COP-LMR 1	0.021	1.822	1.096–3.027	0.116	1.347	0.929–1.951
COP-LMR 2	0.003	2.464	1.373–4.421	0.007	1.902	1.194–3.028

CI: confidence interval; COP-LMR: combination of platelet and lymphocyte to monocyte ratio; CRP: c-reactive protein; HR: hazard ratio; OS: overall survival; PFS: progression free survival.

**Fig 2:**
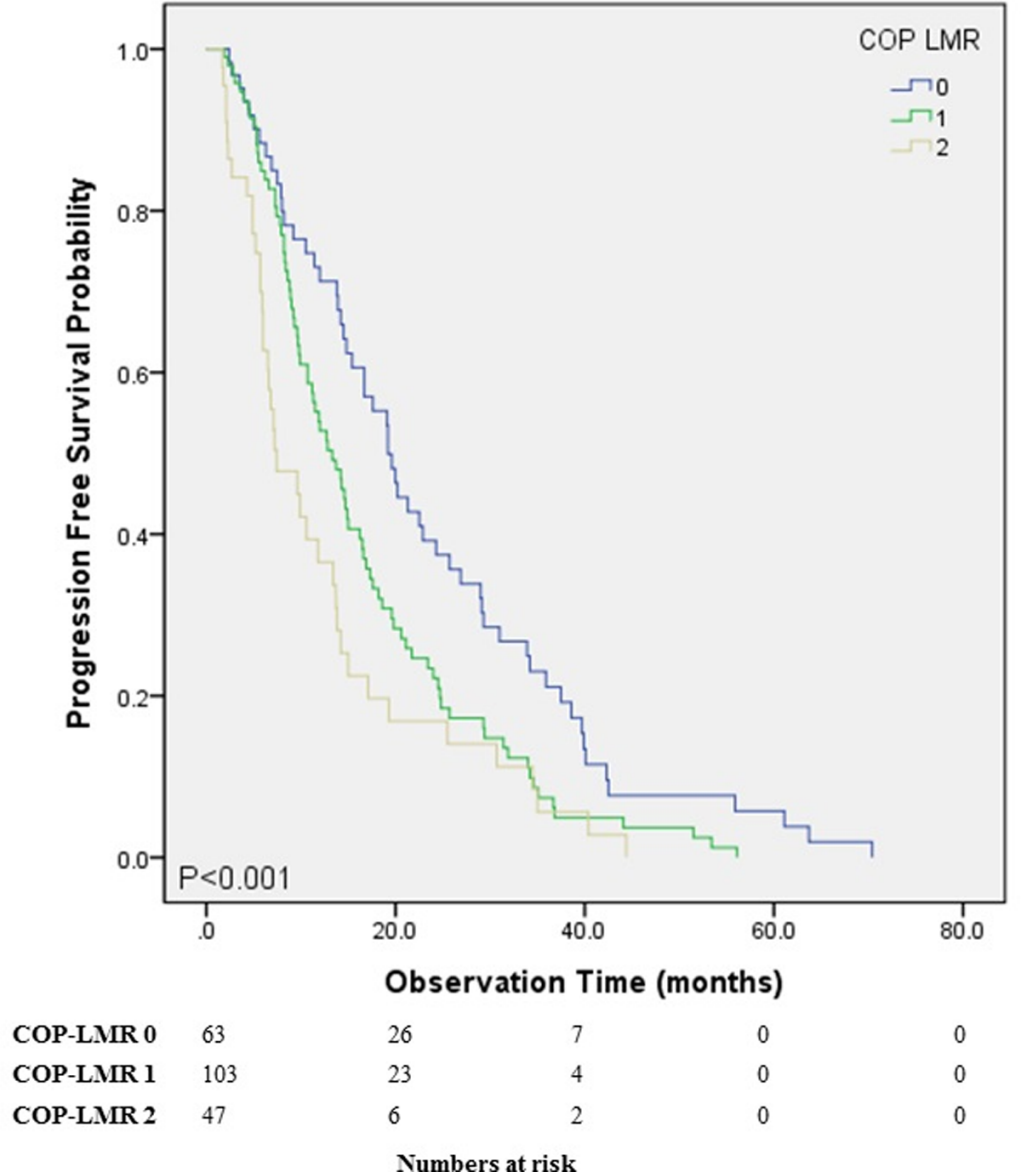
Progression free survival of the stage IV NSCLC patients. PFS between the different COP LMR groups (P<0.001). *COP-LMR*: *combination of platelet and lymphocyte to monocyte ratio; PFS*: *progression free survival*.

Fig 1C shows graphs from Kaplan-Meier analysis assessing predictive value of COP-LMR for OS. There were significant (*P* < 0.001) differences in OS among the three COP-LMR groups. Statistically significant (*P* < 0.001) differences in PFS among the three COP-LMR were also found (Fig 2). Between COP LMR groups 0 vs. 1 and 1 vs. 2, OS showed significant differences (*P* < 0.001 and *P* = 0.023, respectively). OS and PFS decreased as COP-LMR group changed from 0 to 2.

### NSCLC patients without driver mutation

**Fig 3:**
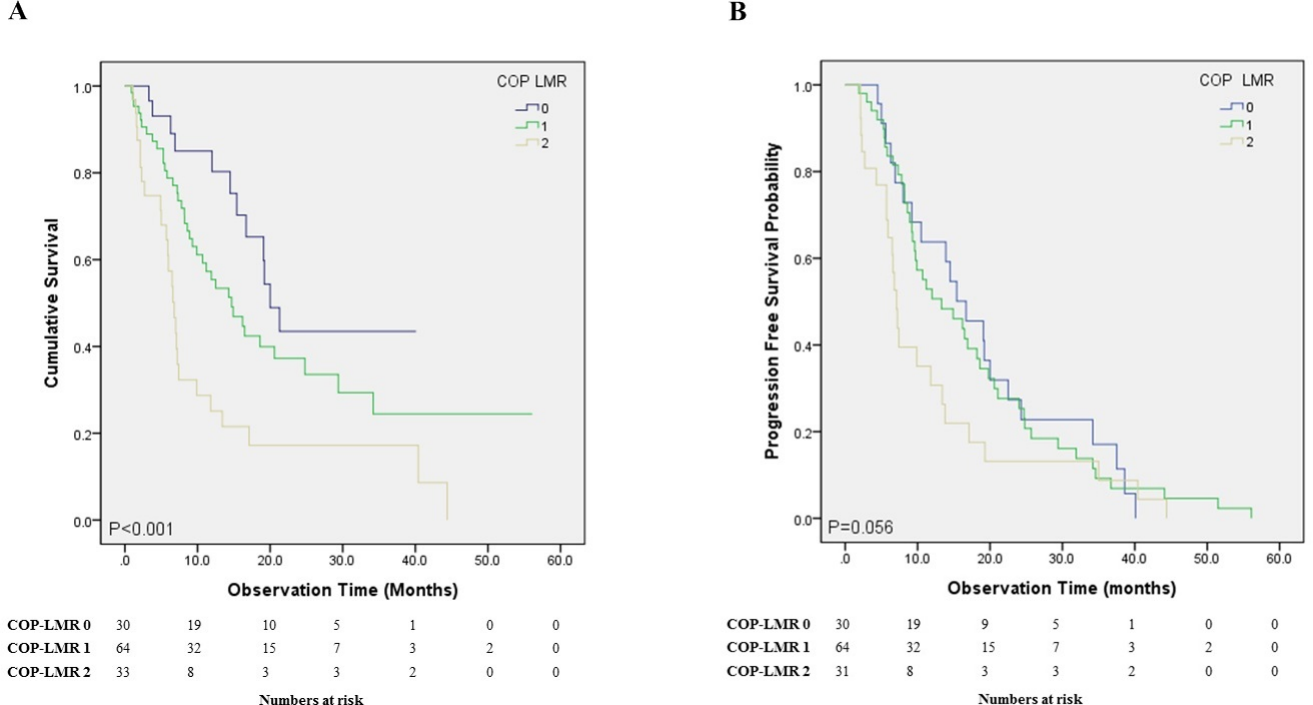
Overall survival and progression free survival of the stage IV NSCLC patients without positive driver mutations between the different COP LMR groups. **A:** OS showed significant difference (P = 0.001) between COP-LMR groups 1 and 2. OS showed no significant difference between COP-LMR groups 0 and 1 (P = 0.071); **B:** PFS showed decreasing tendency as the COP-LMR score is higher, however with no statistical significance (P = 0.056). *COP LMR*: *combination of platelet and lymphocyte to monocyte ratio; NSCLC*: *non-small cell lung cancer; OS*: *overall survival; PFS*: *progression free survival*.

Among 127 NSCLC patients without positive driver mutations, Kaplan-Meier analysis showed significant (*P* < 0.001) difference in OS among three COP-LMR groups (Fig 3A), but not in PFS (*P* = 0.056) (Fig 3B). Median survival times for COP-LMR groups 0, 1 and 2 were 20.0, 14.7 and 6.8 months, respectively. Between COP-LMR groups 1 and 2, OS showed significant difference (*P* = 0.001). However, OS was not significantly different between COP-LMR groups 0 and 1 (*P* = 0.071).

## Discussion

Our results showed that COP-LMR was an independent predictor for shorter overall survival and progression free survival in stage IV NSCLC. Liu et al. have shown that COP-LMR has prognostic value in patients with NSCLC who underwent curative resection [21]. On the other hand, our study population were comprised of stage IV NSCLC patients with MPE. Our results showed that a combination of pretreatment LMR and platelet levels also had prognostic value in more advanced NSCLC.

Thrombocytosis is associated with worse outcomes in NSCLC [19, 26, 27]. Past studies have shown that platelets have significant roles in tumor activity. Platelet is a source of various proangiogenic and anti-angiogenic proteins [28]. It secretes vascular epidermal growth factor (VEGF), transforming growth factor beta (TGF-β) and platelet-derived growth factor (PDGF) that contribute to cancer metastasis, tumor cell invasion, migration and arrest within blood vessels [29–31].

PDGF promotes epithelial to mesenchymal transition by activating Smad and NF-kB pathways, which in turn promote tumor metastasis [32]. On the other hand, tumor cells induce differentiation of megakaryocytes to platelets and proliferation of platelets [33]. Furthermore, tumor cells induce aggregation of platelets to evade immune surveillance [34]. Hence, platelet counts are associated with cancer progression. The cutoff value of platelet counts has not been clearly defined yet, although platelet count of 30.0 x 10^4^ mm^-3^ is often used in previous publications on prognostic value of platelet in cancer [21, 35]. Consistent with these previous publications, the cutoff value of platelet count in our study was also 30.0 x 10^4^ mm^-3^.

It has been suggested that LMR has prognostic value in advanced lung cancer patients who received platinum-based chemotherapies [36] and in advanced-stage EGFR-mutant NSCLC receiving EGFR-TKIs [37]. Furthermore, LMR is an independent prognostic factor in NSCLC who underwent complete resection [18]. In lung cancer, cytotoxic T lymphocytes play a crucial role in anticancer activity [38]. Macrophages are involved in tumor growth through immunosuppression tumor angiogenesis [39] and metastasis [40]. Recruitment of macrophages to tumor site is associated with poor prognosis in various cancers [41]. While the cutoff value of LMR separating high and low groups has not been set, the cutoff value of LMR can influence its predictability. In the present study, cutoff of LMR was 2.47. LMR cutoff values used in previous studies varied from 3.29 to 4.56 [18, 21, 37], higher than the cutoff in the present study. The relatively low cutoff of LMR in our study could be due to the exclusion of patients who had concurrent infections requiring antibiotics therapy.

Both platelet and LMR play a key role in cancer progression. Derived from these two factors, COP-LMR can increase the predictive value in lung cancer patients by combining unfavorable effects and lessening the impact of bias which could happen if only one factor is used.

In the present study, multiple clinical variables were compared among three COP-LMR groups (0, 1 and 2). OS and PFS gradually decreased as COP-LMR changed from group 0 to group 2. Furthermore, OS was significantly shorter in the higher score group when two contiguous groups were compared (0 vs. 1 and 1 vs. 2). There were significant differences in WBC count, hemoglobin, platelet, albumin and CRP levels among these three groups. WBC count, hemoglobin, platelet and CRP levels are associated with poor prognosis in lung cancer [24, 25], while low hemoglobin and albumin reflect poor nutritional status associated with cachexia in cancer patients [12]. It is rational to assert that COP-LMR can categorize NSCLC patients into independent groups in terms of prognosis.

We also noted that the increase in squamous cancer portion and the increase in COP-LMR show positive relation. From the previous study on COP-LMR, the same clinical correlation is also shown [21]. We assume that squamous lung cancer could have accompanied more active systemic inflammation compared to non-squamous NSCLC. However further study with larger study population is necessary to confirm the assumption.

In comparison with a previous study [21], we tried to minimize nonmalignant factors that could influence levels of platelet and LMR. We excluded patients who had infections requiring antibiotics treatment such as bacterial pneumonia and pulmonary tuberculosis at the time of diagnosis of lung cancer. Patients with underlying hematologic diseases were also excluded.

This study has a few limitations. First, due to the retrospective nature of this study, selection bias or unidentified factors might have influenced outcomes. However, we consecutively collected data from six centers in order to minimize selection bias. Second, prevalence of driver mutations among study patients, mostly EGFR mutation, was high (41.5%) compared to that in other advanced NSCLC populations [42]. However, this proportion is not unusual in Asian NSCLC populations [43, 44]. Furthermore, Kaplan Meier curve analysis of NSCLC patients without driver mutations showed significant difference in OS among the three COP-LMR groups (Fig 3A).

## Conclusions

A combination of pretreatment LMR and platelet levels can be used to predict short survival in stage IV NSCLC patients who underwent active anticancer treatment. Further research is needed to elucidate the underlying pathophysiological background for predictability of combined inflammatory biomarkers.

## Supporting information

S1 File Study patients’ dataset. (XLSX)
